# *Notes from the Field:* Wound Botulism Outbreak Among a Group of Persons Who Inject Drugs — Dallas, Texas, 2020

**DOI:** 10.15585/mmwr.mm7115a3

**Published:** 2022-04-15

**Authors:** Leslie D. Edwards, Ivorry Gomez, Suzanne Wada, Erin M. Swaney, Michelle B. Caruthers, Irina Cody, Farrell A. Tobolowsky, Janet Dykes, Laura Ford, Kenneth R. Davis, Chelsey T. Griffin, Wendy Chung

**Affiliations:** ^1^Division of Foodborne, Waterborne, and Environmental Diseases, National Center for Emerging and Zoonotic Infectious Diseases, CDC; ^2^Acute Communicable Disease Epidemiology Division, Dallas County Department of Health and Human Services, Dallas, Texas; ^3^Public Health Laboratory, Texas Department of State Health Services; ^4^Emerging and Acute Infectious Disease Unit, Texas Department of State Health Services; ^5^Epidemic Intelligence Service, CDC.

On December 9, 2020, Dallas County Health and Human Services (DCHHS) was notified of a hospitalized male, aged 33 years (patient A), who was experiencing homelessness and had bilateral ptosis, upper and lower extremity weakness, and respiratory failure requiring intubation. The patient reported injecting methamphetamines, and physical examination noted track marks but no overt skin wounds or abscesses. Patient A was treated with naloxone. Heroin and methamphetamines were detected in the patient’s urine. Myasthenia gravis was initially suspected; however, botulism was considered when the patient did not respond to treatment with pyridostigmine and steroids and the patient’s weakness continued to progress. DCHHS contacted the Texas Department of State Health Services (DSHS) and CDC’s botulism clinical consultation service.* Heptavalent botulinum antitoxin (BAT) was released by CDC on December 9, 2020, and administered to the patient on December 10. Botulism testing results were not available before treatment with BAT. Botulism neurotoxin (BoNT) types A and B were detected in the patient’s serum specimen using the BoNT Endopep-MS assay (*1*).

On December 18, 2020, DCHHS was notified of a female aged 39 years (patient B), admitted to a different hospital with difficulty swallowing during the past 2 weeks and respiratory failure requiring intubation. Patient B was treated with naloxone. Multiple chronic and several fresh wounds were noted during the physical examination. BAT was administered, and BoNT types A and B were detected in this patient’s serum specimen. Acquaintances of patient B reported injecting black tar heroin subcutaneously (skin popping) and sharing this drug with patient A. 

Interviews with acquaintances of patient B identified three additional persons (patients C, D, and E) who injected drugs and were admitted to the hospital during December 2–21, 2020, with cranial nerve impairment including diplopia (two), blurred vision (two), bilateral ptosis (one), upper extremity weakness (two), and respiratory failure requiring intubation (two). Patient D was 13 weeks pregnant at the time of hospitalization and left the hospital against medical advice 10 weeks after receiving BAT. Patient E was located and brought to the hospital by family members following health department outreach and left against medical advice immediately after receiving BAT. In January 2021, two persons (patients F and G) with diplopia, blurred vision, and shortness of breath who had injected drugs with either patient B or patient E were concerned that they might have botulism and were evaluated at area hospitals. All seven patients identified in this investigation were treated with BAT. Four (57%) patients required mechanical ventilation and prolonged intensive care.

Serum specimen collected from all seven patients before administration of BAT were tested for BoNT; patients C, D, E, F, and G received a negative test result and were classified as having probable wound botulism cases for surveillance purposes.^†^ Three serum specimens were not maintained at proper temperature during shipping, which might have affected testing results. Stool cultures from patient D yielded positive test results for *Clostridium botulinum* type A using the BoNT Endopep-MS assay, raising the question of whether this patient was part of the wound botulism outbreak or had foodborne botulism.

This is the first wound botulism outbreak reported in Texas and the largest in the United States outside of California (*2*,*3*). During 2010–2019, a total of 206 laboratory-confirmed cases of wound botulism were reported in the United States, including 160 (78%) in California and eight (4%) in Texas.^§^ The rarity of reported wound botulism outbreaks might be partially related to challenges from stigma precluding identification and epidemiologic linkage of patients who injected drugs together or purchased drugs from the same source. In this outbreak, public health officials discovered additional cases by interviewing patient acquaintances who had also injected drugs and were aware of the early signs and symptoms of botulism. Case-finding efforts could be improved if clinicians ask patients with suspected wound botulism whether they have acquaintances with symptoms and whether syringe exchange service programs share wound botulism educational materials with clients. Increased awareness of wound botulism among patients with cranial nerve impairment and progressive weakness, and among persons who inject drugs outside of California, might also help to identify additional cases. 

## Footnotes

*
https://www.cdc.gov/botulism/health-professional.html#:~:text=If%20you%20suspect%20your%20patient,at%20770%2D488%2D7100

†
https://ndc.services.cdc.gov/case-definitions/botulism-2011/#:~:text=Botulism%2C%20wound-,Clinical%20Description,Symmetric%20paralysis%20may%20progress%20rapidly

§ https://www.cdc.gov/botulism/surveillance.html
